# Awareness Mentality and Strategic Behavior in Science

**DOI:** 10.3389/frma.2021.703159

**Published:** 2021-08-06

**Authors:** Rafael Ball

**Affiliations:** Director ETH Library, ETH Zurich, Zurich, Switzerland

**Keywords:** scholarly communication, scientometrics, awareness, bibliometrics, academic publishing

## Abstract

Acknowledgement of scientific achievements was and is essentially achieved through the citation of a publication. Increasingly, however, it is no longer just the publication itself that plays an important role, but also the degree of attention that a scientist achieves with this very publication. Thus, the importance of strategic behavior in science is progressing and an awareness mentality is spreading. In this paper, the causes and backgrounds of this development are discussed, identifying the use of reductionist, quantitative systems in science management and research funding, the loss of critical judgment and technocratic dominance, quantitative assessments used for decision making, altmetrics and the like as alternative views, the use of perception scores in reference databases and universities as well as ambitions of journals as main drivers. Besides, different forms of strategic behavior in science and the resulting consequences and impacts are being highlighted.

## Introduction

The number of scientific publications has been increasing dramatically for decades. Every day 8,500 scientific papers are being published and annually there is an increase of three million papers evaluated in the database “Web of Science” alone, which accounts for only 5% of the scientific journal output (Oeser 1976, 121). The analysis of peer-reviewed Science and Engineering publications in the Scopus database as of July 2017 show an annual growth of 3.9% per year worldwide. This leads to a doubling of total publications from 1.5 million in 2006 to almost three million publications in 2016 (National Science Foundation 2018, 101). Large platforms such as academia.edu multiply the amount of information through the second and third publication of publications, as do the countless institutional and discipline preprint or postprint repositories (Conard, 2018, 255). The duplication of content alone because of digital dissemination possibilities leads to an ever-increasing amount of scholarly information competing for the limited attention of scholars. As a consequence of the dramatically increasing amount of information (Meadows, 1998, 15–16; Proquest, 2020) and the almost unimprovable attention capacity of human beings, there is an ever-increasing perception deficit, which in turn must be compensated for by a wide variety of awareness measures on the part of researchers. Not every publication is noticed and cited any more. A competition for attention has arisen, which is conducted by various means. These increasingly include the communication tools of social media. At the same time, proving that one’s own research and output are perceived is strategically relevant, especially for young researchers, for their scientific and personal survival in the academic world in the competitive struggle for funding and positions. An awareness mentality is emerging in science paired with and at the same time as an expression of strategic behavior. As long as citation numbers and other quantitative metrics were only an end in themselves for one’s own perception analysis, they could not do any major damage. Adapted behavior only became problematic and necessary in the sense of a “survival strategy” when publication figures, citation numbers, the allocation of research funding, personal careers and the professional future of academics combined to form a dangerous amalgam. “Academia has become a publish or perish world” (Moosa, 2018, 2).

This strategic behavior serves as a survival strategy in academia today. Thus, I argue that only those who behave strategically in terms of the awareness mentality have a long-term chance of survival in the academic ecosystem.

## Delimitation and Definition of Terms

### The “Awareness Mentality”

In the context of this publication I use the terms “awareness” and “awareness mentality” to mean attention, perception, but also awareness and knowledge or familiarity. In other words, a mentality whose guiding principle is the generation of attention, of perception and—if we break it down to the individual scientist—of the creation of his or her own perception in the sense of being perceived and made known. “Scientists trying to maximise attention must not only care about selling their product, they must also care about making it a stir” (Franck, 2002, 8). In this means, I claim—and then try to prove and explain—that today’s scientist invests a large part of his or her labor in generating awareness. Or must invest. This strategic pressure to adapt leads to an inner and outer attitude that I call “awareness mentality.”

### Strategic Behavior in Science

A person behaves strategically when he or she is guided by internal or external goals and—since we are talking about scientific behavior—subordinates content, questions, research design, methods, and communication of results to these goals. This may be for example, be conducted by means of “salami sclicing”—e.g., publishing an excessive number of papers from a single study–as discussed by Bailey (2002). This does not correspond to the idea and principles of academic science. Scientists, in the self-referential system of science, which defines goals and questions from within itself, should not be guided by external goals that are not meant to be self-referential in the sense of science (Rheinberger, 2018). This outlines the basic topics of this article. Accordingly, it would still have to be clarified why scientists allow themselves to be guided (or have to be guided) by goals other than those intrinsic to science and by what methods and means they do so.

## A Short Look at the History of Science Communication

Starting with the last question I try to deduce what may have caused the claimed awareness mentality and how it is shaping in the context of changes in scholarly communication. To do this, we take a brief look at the history of scholarly communication.

We see three paradigm shifts in scholarly communication (Ball, 2020): About 2000 years ago, there was the shift from oral to written communication, whose strongest representatives were Plato and Aristotle. After the triumph of written communication and its externalization in the most diverse forms dominated by the respective description and media techniques, the invention of letterpress printing with movable type by Johannes Gutenberg in 1,452 is the second paradigm shift in scholarly communication. It was the first time that scholarly communication became a mass topic and the dissemination of large quantities of identical texts was made possible independently of geography and time. Both the Reformation and the Renaissance of science were children of printing (Greenblatt, 2012). The third paradigm shift is the emergence of mass digitization, which radically has changed everything—from the creation of content and its production, to its dissemination and archiving.

From now on, it was possible to disseminate scientific content even faster in even greater mass and reach, but at the same time more freely and independently of the established systems of the print era. The awareness mentality picked up speed at the same time as digitality, even though the topic of awareness already had its beginnings in the pre-digital era, especially after the emergence of mass (or big) science after the end of World War II (Price and Derek, 1963).

The notion of awareness outlined here does not (or not primarily) refer to the marketing of one’s own scientist persona, but to the marketing of his or her publications. This was and is the central method of drawing attention to one’s own successes and qualities (Franck, 2002, 4). The currency of science has always been attention and recognition for the creation of new knowledge (Tunger, 2018). The publication and its perception created and still creates recognition. We still define four central functions of a publication today (Shorley and Jubb, 2013): registration, thanks to which the scientific findings are protected by copyright and can be cited at the same time. Certification, which proves that it is a contribution of impeccable quality (for example, through peer reviewing). Perception, which draws the attention of other scientists to one’s own findings and makes them available as the subject of further research, and archiving, which guarantees long-term storage and accessibility of scientific findings for posterity.

These four functions are still fulfilled by publications in times of digitality, but today they are weighted differently and implemented through new, digital methods.

## Ratings and Rankings in Science

Acknowledgement of scientific achievements was and is essentially achieved through the citation of a publication. Through professionalization and automatic processing in citation indices, this form of awareness has led to a quantification of scientific results in which it is increasingly no longer the content of the publication that matters but the degree to which it is being perceived. This has transformed the quality assessment of content into a measurement of its perception. The run on citation and measurement metrics in science represents the beginning of an awareness mentality that subsequently has become a veritable mass movement through digital systems. The system of quantitative perception measurement is about to take on a life of its own, when successful science is no longer defined as “Being good,” but as “Looking good.”

Digitality enables completely new forms of production, distribution and screening of scientific content. This is the technical perspective. But also, in terms of the sociology of science, the perception of results has become an increasingly important aspect of scientific activity. Producers of scientific content are adapting to this and start acting strategically. They will and must prefer forms and formats that guarantee them the appropriate attention (Weingart, 2005, 331). These are no longer only classic journal articles, books, and conference papers. Instead, contributions in the various channels of social media and other, new, digital network formats are increasing (Weingart, 2005, 272). The change in output formats is multifactorial. New technical potentials play just as much a role as normative elements, such as the demand for open access and open science, as well as competitive pressure in career planning or the financing of positions and funding (Krull, 2017, 46).

The increasing importance of rankings and ratings of individuals, institutions and countries is used and loved by the scientific community but at the same time is being criticized and rejected. Today, there is an almost unmanageable number of indicators for evaluating publication activity and its perception (Hinz et al., 2020). This includes not only the classic citation indicators, but also increasingly alternative metrics (Haustein et al., 2014), such as those that have been collected since 2010 and, above all, depict perception in social media: “The composition of the attention score is based on an algorithm that adds up the attention of scientific output in the various sources, weighted differently” (Tunger et al., 2017, 6).

The fact alone that such perception scores are already found extensively not only on scientists’ websites, but also in the established reference databases of scientific literature such as the Web of Science, Scopus or Dimensions, as well as in the repositories of universities, shows that researchers cannot avoid quantification when competing for attention and awareness.

Although “in general (...) altmetrics (should) not be seen as a substitute for classical peer review in the context of quality assessment of scientific output, but are interpreted as a way to get a second opinion and additional information” (Tunger et al., 2017, 7), the new attention scores are also and increasingly significant and must be used by researchers. It is a tightrope walk to establish helpful instruments in science and, at the same time, not to pave the way for quick incentives.

Thus, there is a tension between, on the one hand, meaningful indicators that can help researchers measure the impact of their research output. On the other hand, these same indicators put even more pressure on researchers to design their work in such a way that they achieve satisfactory values. Breaking out of this vicious circle is practically impossible, especially for young scientific careers. Quantitative assessments of publication performance are used as central decision-making aids in the allocation of funds and positions (Osterloh and Frey, 2015, 65).

With this practice in science and publication management, we should therefore not be surprised today at the flood of publications, the rising journal and APC prices or the use of reductionist, quantitative systems in science management and research funding, nor at the loss of critical judgement and the marching through of technocrats (Andersson, 2008, 16).

## Causes of Strategic Behavior in Science

The causes for strategic (mis) behavior and thus for the attempt to gain more attention and more citations are manifold, even if they have not yet been researched in detail (Huberts, 2014).

Essentially, the causes are to be found in the pressure of the overall system of science, including its culture of communication and publication. Certainly, questions of personal responsibility and morality also play a role since there are also people among scientists who do not take the truth very seriously. The coupling of the number and quality of publications (and thus perceptions) with the awarding of funding and career options is symptomatic and puts scientists under enormous pressure (Hall and Martin, 2019, 414). The system sets false incentives and causes despair, fear of losing one’s job and livelihood, career crumbling, and the fear of not being awarded funding.

But the expectations of scientific journals are also increasingly rising. In the last 10 years, the number of rejected article submissions has increased tenfold (Hall and Martin, 2019, 414). Under pressure from editors (who in turn are under pressure from publishers), journals must publish ever more spectacular findings and reports. Competition among journals and the assessment of their importance and quality through performance indicators and awareness also lead to fierce competition in the publishing industry. This is passed on to the authors and puts additional pressure on scientists.

False incentives in the reward system of science cause extremely high expectations of results in Asia, for example. In particular, the expectation of the social elites concerning visible success of science in their countries is the cause of enormous personal pressure on researchers and one of the causes of questionable publications from Asia (Lee and Schrank, 2010).

## Various Forms of Strategic Behavior in Science

Strategic behavior in science can take many different forms: You can subordinate research to trendy topics and thus try to obtain as much funding as possible, you can carry out spectacular experiments with the attempt to achieve spectacular results. Or you can optimize your citation rate through the strategic selection of the publication media. “Negative results,” failed experiments or hypotheses that cannot be confirmed, on the other hand, are neither desired in the scientific publication system, nor do they achieve the necessary acceptance and attention, nor do they survive the peer review process (ScienceMatters, 2021).

None of this constitutes scientific misconduct. In the sense of increasing success and optimizing awareness, it is morally questionable in the worst case, but not reprehensible (if the category of morality may be applied here at all). Nevertheless, the strategy of achieving (or having to achieve) scientific success through increased awareness leads, in the age of digitality, to a situation where attention is no longer to be achieved solely through the classic citation metrics, but increasingly through the systems of social media, which operate in a short-lived, fast, high-frequency, and non-stop manner (Barth, 2019, 9). Thus, the system requires scientists today not only to have in-depth knowledge of social media and their use, but also to adequately prepare appropriate content, which differs significantly from classic publications, especially in the scope and depth of the argumentation (Ram and Rameshwar, 2016, 229).

It is only a very small step from a purely objective, reserved, research, and knowledge-driven science to a working manner that formulates questions, designs research experiments, and presents results in such a way that it primarily serves to generate attention. However, not everyone who uses social media skillfully and thus makes his or her research better known than through pure “citation perception” is conducting strategically controlled or even dubious research.

Strategic action, scientific misconduct, and manipulation move close together in a narrow corridor. The more pressure there is on researchers to generate attention for their results, the faster the distinction between the two becomes blurred. If “Looking good” is better rewarded than “Being good,” the inhibition threshold for the step from knowledge-driven science to an attention-seeking show decreases. And with it the quality of research, whose results are increasingly no longer reproducible, which causes the already discussed “crisis of reproducibility,” or at least exacerbates it (Moosa, 2018, 71–73).

Questionable and inappropriate behavior in science is becoming increasingly common. In a survey conducted by Bouter (2015), 43% of all researchers admitted to have published questionable data and results, 2% even to have deliberately falsified. When asked about their opinion of other scientists, it was suspected that 14% of others falsify and 72% publish questionable results.

From careless editing of data to deliberate fraud, all forms of manipulation are being demonstrated. Boundaries are being extended, hypotheses are being adapted to the results and vice versa. Methods are being falsified or rearranged, as is the underlying data. Other phenomena observed are text recycling, self-plagiarism and genuine plagiarism (Öchsner, 2013, 95).

It is reasonable to assume that at least a certain proportion of such wrongdoing can be traced back to an exaggerated or mislead form of awareness mentality. Against this background the inappropriate behavior in science can be understood as an expression of the aim to gain attention and secure livelihoods.

In one study a significant increase in titles of scientific publications in medicine, life sciences and physics that end with a question mark is shown (Ball, 2007). For example, in medicine, the proportion of “question mark articles” increased eightfold in the study period. A random qualitative analysis showed that scientists increasingly chose daring or spectacular titles for their publications to attract attention. To nevertheless remain scientifically credible, the titles are ended with a question mark, so that there is still the option of retraction–just in case.

This in turn leads to some challenges, particularly related to peer reviewing. On the one hand, scientific misconduct can simply not always be identified in a peer review process—especially not when we are in the gray area between strategic behavior to attract attention and actual misconduct. This reduces the assurance of certification, which as one of the four basic functions of a publication mentioned above should also be achieved through peer reviewing.

Another challenge of peer reviewing is the already mentioned fact that negative results often do not survive the peer reviewing process. If this does not change in the future, it is to be feared that peer reviewing will fuel a misunderstood and exaggerated form of awareness mentality.

## Impacts and Consequences

The four basic functions of a publication (Shorley and Jubb, 2013) have not become meaningless even in the age of digitality, social media and awareness hype. The individual scientist must still fulfil the basic functions of a publication. However, it is no longer enough to send the manuscript to a publisher and to wait for the paper to be accepted: This is only the basic step of a publication practice that has changed fundamentally. Today, proof must also be provided that the paper is available in Open Access. Today, depositing the paper in an institutional or subject-specific repository is just as much an obligation as supplementing the article with the research data and references used, as well as linking it in various academic networks. Blog posts about new findings of the paper are expected, as is the use of Twitter and other social media to draw attention to the new paper. Marketing for the purpose of generating awareness has an increasing share in the publication effort. In addition, the pure academic community and the interested public are increasingly merging. If you really want to attract attention to yourself and your research, another need is to present and explain your results in a generally understandable way. In doing so, the results not only serve their own specialist community, but also a general audience. Video messages and Instagram appearances complete the awareness campaign.

In a possible perspective of scientific publishing, we have to state that the classical concept of publication with all its implications will dissolve if the success of a publication is no longer measured only by the truthfulness of the messages, but by the mere determination and quantification of the perception of what is communicated (and no longer the perception itself). If perception (and its ascertainment) becomes to be more important than truth, then all barriers will fall for the uncontradicted boundary shift from knowledge to opinion and vice versa. “Looking Good” becomes more important than “Being Good,” also because a rapidly increasing number of publications makes qualified reception impossible. If scientific results are then increasingly no longer reproducible, the common sense of the basic principle of publishing scientific findings for the purpose of their reception, discussion and further development will finally go bankrupt.

## Data Availability

The original contributions presented in the study are included in the article/supplementary material, further inquiries can be directed to the corresponding author.
